# Induced abortion according to immigrants’ birthplace: a population-based cohort study

**DOI:** 10.1186/s12978-020-00982-z

**Published:** 2020-09-14

**Authors:** Susitha Wanigaratne, Mei-ling Wiedmeyer, Hilary K. Brown, Astrid Guttmann, Marcelo L. Urquia

**Affiliations:** 1 ICES, Toronto, Ontario Canada; 2 MAP Centre for Urban Health Solutions, Unity Health, Toronto, Ontario Canada; 3 Child Health Evaluative Sciences, The Hospital for Sick Children, Toronto, Ontario Canada; 4 BC Women’s Hospital and Health Centre, Vancouver, British Colombia Canada; 5 Centre for Gender and Sexual Health Equity, Vancouver, British Columbia Canada; 6 Interdisciplinary Centre for Health & Society, University of Toronto Scarborough, Toronto, Ontario Canada; 7 Women’s College Research Institute, Women’s College Hospital, Toronto, Ontario Canada; 8 Dalla Lana School of Public Health, University of Toronto, Toronto, Ontario Canada; 9 Department of Paediatrics, Hospital for Sick Children, University of Toronto, Toronto, Ontario Canada; 10 Manitoba Centre for Health Policy, University of Manitoba, Winnipeg, Manitoba Canada

**Keywords:** Abortion, induced, Reproductive health, Epidemiology, Emigrants and immigrants, Health equity

## Abstract

**Background:**

Most abortions occur due to unintended pregnancy. Unintended pregnancies are linked to poor health outcomes. Canada receives immigrants from countries with disparate sexual and reproductive health contexts which may influence abortion rates post-migration. We examined the association between abortion and region of birth and birth order among Canadian immigrants.

**Methods:**

We conducted a population-based person-years (PY) cohort study in Ontario, Canada using administrative immigration (1991–2012) and health care data (1991–2013). Associations between induced abortion and an immigrant’s region of birth were estimated using poisson regression. Rate ratios were adjusted for age, landing year, education, neighborhood income quintile and refugee status and stratified by birth order within regions.

**Results:**

Immigrants born in almost all world regions (*N* = 846,444) were 2–5 times more likely to have an induced abortion vs. those born in the US/Northern & Western Europe/Australia & New Zealand (0.92 per 100 PY, 95% CI 0.89–0.95). Caribbean (Adjusted Rate Ratio [ARR] = 4.71, 95% CI 4.55–4.87), West/Middle/East African (ARR = 3.38, 95% CI 3.26–3.50) and South American (ARR = 3.20, 95% CI 3.09–3.32) immigrants were most likely to have an abortion. Most immigrants were less likely to have an abortion after vs. prior to their 1st birth, except South Asian immigrants (RR = 1.60, 95% CI 1.54–1.66; RR = 2.23, 95% CI 2.12–2.36 for 2nd and 3rd vs 1st birth, respectively). Secondary analyses included further stratifying regional models by year, age, education, income quintile and refugee status.

**Conclusions:**

Induced abortion varies considerably by both region of birth and birth order among immigrants in Ontario.

## Plain English summary

Introduction – Most abortions occur due to unintended pregnancy. Unintended pregnancies are linked to poor health outcomes. Immigrants to Canada come from countries with varying access to sexual and reproductive health care which may influence abortion rates. We examined the relationship between abortion and region of birth and birth order among Canadian immigrants.

Methodology - We conducted a study in Ontario, Canada using official immigration (1991–2012) and health care data (1991–2013). The relationship between abortion with an immigrant’s region of birth and number of previous children were examined.

Results - Immigrants born in almost all world regions were 2–5 times more likely to have an induced abortion vs. those born in the US/Northern & Western Europe/Australia & New Zealand. Caribbean, West/Middle/East African and South American immigrants were most likely to have an abortion. Most immigrants were less likely to have an abortion after vs. prior to their 1st birth, except South Asian immigrants. Secondary analyses included further stratifying regional models by year, age, education, income quintile and refugee status.

Conclusion - Rates of induced abortion are considerably different according to both region of birth and birth order among immigrants in Ontario.

## Background

Over half of pregnancies in the United States are intended (i.e., desired then or sooner) of which 3% end in abortion [1], while the remaining 45% of pregnancies are unintended with 42% ending in abortion [2]. In Canada 61% of women report having at least one unintended pregnancy [3]. Given unintended pregnancies are linked to a myriad of negative health [4], economic and social circumstances [5], access to abortion is a critical component of sexual and reproductive health care. The path from conception to abortion is complex and can include consensual or non-consensual sexual activity, contraceptive non-use, ineffective use or failure, unintended pregnancy, and the decision to terminate [6]. Parts of this path have societal, cultural, religious, [7] economic, legal or systemic determinants which contribute to abortion decisions.

Few studies have examined induced abortions among immigrants, and none could be found in Canada, where abortions have been publicly funded since 1988 and can be obtained without restriction (i.e., within gestational limits, with variation in accessibility by geography [8]). There are many factors that could influence immigrant women’s use of abortion services after migration. In the last 45 years, immigrants to Canada have largely come from “developing” countries where there are full or partial restrictions on abortion [9]. Contraceptive prevalence rates rose in developing countries between 1990 and 2010 but were highly variable (82% in East Asia and 15% in West Africa) [10]. Exposures to these varying pre-migration sexual and reproductive heath and socio-cultural contexts may continue to shape understanding and decisions post-migration [11]. In the Canadian post-migration context, modern contraceptive prevalence is relatively high (~ 74% between 1990 and 2010 [10]) and access to primary health care services is publicly funded; however immigrants in Ontario are less likely to be enrolled in any enhanced primary care model and particularly those which are high quality, continuous and comprehensive [12] which likely limits access to responsive and flexible sexual and reproductive health care. A recent Australian systematic review identified numerous barriers to effective sexual and reproductive health care among immigrants [13]. Given very different sexual and reproductive health contexts and experiences prior to and after arrival, epidemiologic studies are needed to better understand patterns of induced abortion among immigrants in Canada.

The purpose of this study is to examine induced abortions to inform efforts to reduce unintended pregnancies and their negative consequences among Canada’s large and diverse immigrant population. We used population-based administrative databases to estimate rates of induced abortion according to world region of birth for all immigrant females. Since the need for contraception and family planning varies over the life course and helps to achieve desired family size which may vary by region of birth, we also estimated induced abortion rates by birth order among females who gave birth in Ontario.

In this study, “immigrants” refer to refugee and non-refugee immigrants who were successful in gaining legal permanent residency status in Canada and who have access to provincially funded health care services at arrival (refugees) or within 3 months of arrival (non-refugee immigrants). The setting of this study is the province of Ontario, the most populous province in Canada (14 million) with the highest number and proportion of immigrants, 70% of whom arrived between 1985 and 2016. Permanent resident immigrants are generally categorized into three streams [14] – economic, family and refugee class; 60, 27 and 11% of all immigrants respectively [15]. Briefly, economic immigrants are selected based on skills and their ability to contribute to Canada’s economy; family class immigrants are sponsored by adult family members who are either Canadian citizens or a permanent residents living in Canada; and refugees are those who unable or unwilling to return to their country of origin owing to a well-founded fear of being persecuted for reasons of race, religion, nationality or membership in a particular social group.

## Methods

Population-based linked administrative databases were accessed at ICES (formerly known as the Institute for Clinical Evaluative Sciences) in Toronto, Ontario to conduct this retrospective study. ICES is an independent, non-profit research institute whose legal status under Ontario’s health information privacy law allows it to collect and analyze health care and demographic data, without consent, for health system evaluation and improvement. ICES houses data on individuals eligible for coverage under the Ontario Health Insurance Plan (OHIP). OHIP provides publicly funded health care coverage at no cost to those with Canadian citizenship, permanent resident status and some temporary residents with work permits.

### Study design and inclusion/exclusion criteria

We took a retrospective longitudinal cohort approach and enumerated all induced abortions occurring since migration (i.e., since receiving permanent resident status) to Canada, measured per 100 person-years (PY) of follow-up time. Given our study design, we did not estimate annual abortion rates per 1000 women, as is typically seen in cross-sectional or repeated cross-sectional studies. Persons who indicated their sex to be female were included in the study if they immigrated to Canada between April 1, 1991 and December 31, 2012, were registered for OHIP and resided and were alive in Ontario for at least one year between 1 April 1991 and 31 March 2014 following arrival. For each year a female met the stated eligibility criteria and was between the ages of 15–44 years, a year of follow up time was assigned and summed in PY. Induced abortions were enumerated for females who contributed a PY of follow-up time in the year abortion(s) occurred. To examine induced abortion rates prior to a birth, the population included those who delivered up to three or more consecutive singleton births in Ontario hospitals between April 1993 and March 2014. Those who did not have all their births in Ontario were excluded from birth order analyses, along with all their children.

This study does not include asylum seekers awaiting a refugee determination hearing which decides their eligibility for permanent residency. Asylum seekers are eligible for federally funded health care services while they wait for their hearing (i.e., Interim Federal Health Program). This study also does not include migrants: with temporary (1 year) work permits who fill Canada’s short-term labour needs and are eligible for provincially funded health care; with temporary student permits who may be eligible for student health care plans; who are undocumented (estimated at ½ million, the majority initially entered Canada legally but have overstayed their permits [16]) who have limited or no access to publicly funded health care.

### Data sources

Several population-based administrative databases were linked to perform this study. These datasets were linked using unique encoded identifiers and analyzed at ICES.

The Ontario portion of the Immigration, Refugee and Citizenship Canada Permanent Resident Database (IRCC-PRD) contains the legal immigration records for all individuals who obtained permanent residency in Canada between January 1985 and December 2012 and intended to reside in Ontario. About 86% of individuals in the Ontario portion of the IRCC-PRD were linked to Ontario’s healthcare registry consisting of Ontarians eligible for publicly funded healthcare insurance in Ontario between April 1, 1990 and March 31, 2014 with a valid health card number. There were small standardized differences (< 0.2) between linked and unlinked individuals across nearly all sociodemographic variables and regions of birth indicating that the linked individuals were largely representative of the original IRCC-PRD [17].

Reporting of induced surgical or pharmacological abortions in publicly funded clinics is mandatory and captured in the Canadian Institute for Health Information’s (CIHI) Discharge Abstract Database (DAD - hospital acute care visits), Same-Day Surgery and the National Ambulatory Care Reporting system (NACRS - emergency department visits). Out-of-hospital abortions are captured using OHIP billing codes for surgical abortions. The CIHI DAD was also used to identify hospital births to immigrant females and their birth characteristics including birth order.

The Office of the Registrar General’s Vital Statistics Death registry (1991–2013), supplemented by mortality recorded in the healthcare registry and other administrative databases was used to identify any deaths among immigrants included in the initial population. For the year in which a death occurred, follow-up time was calculated from January 1 to the date of death that year.

### Variables

Induced abortion (henceforth “abortion”) was defined as any surgical or pharmacologically induced termination of pregnancy in the absence of a diagnosis of spontaneous abortion. Given the single-payer health care system in Ontario and the use of population-based databases (rather than self-reported surveys), we report on all induced abortions except a minority conducted in private clinics. If two abortion procedures were recorded within 40 days of each other for a given woman, they were considered the same event and first abortion date was used. CIHI diagnosis codes and OHIP billing codes for induced abortion are described elsewhere [18]. Late induced abortions were any abortions occurring at 15 weeks or more gestation identified using fee code S785. Late terminations are eligible for publicly funded payment if gestational age is confirmed by ultrasonography. In 2017 Mifesgymiso (i.e., Mifespristone) for pharmacologically induced abortion became available free of charge in Ontario, [19] however no abortions were carried out using this drug during the time period of this study. Medically indicated abortions (i.e., for health reasons) could not be excluded since Canadians do not have to provide a reason for abortion. Consequently, Canadian statistics on abortion reasons could not be found but 2018 data from Florida (USA) indicates ~ 1% of all abortions in that state were due to a life endangering physical condition or serious fetal genetic defect/deformity/abnormality. An additional 3% were performed to protect the physical/emotional/psychological health of the mother (not life endangering); while the remainder were due to rape/incest (< 0.1%), social/economic reasons (20%) or no reason provided was provided (75%) [20].

The exposure of primary interest was region of birth categorized using country of birth as reported in the IRCC-PRD and the United Nations geographical classification system which categorizes countries into 22 sub-regions [21]. Sub-region categories were explored for similarity in induced abortion rates to identify geographically adjacent sub-regions or sub-regions with similar levels of “development” for which aggregation would not mask important heterogeneity. Based on this exploration some sub-regions were aggregated into larger regions.

We also examined abortion rates by birth order which was defined as the complete sequence of up to three consecutive live births from the same mother since there were few females that had more than three births. At the time of each pregnancy the alive status of previous siblings was determined using the vital statistics registry and in-hospital death data. If prior siblings were not alive by the estimated conception date of the current live birth, the birth order was reduced for each subsequent live birth.

Covariates as reported in the IRCC-PRD included: country of birth, age at arrival (continuous and categorized as 0–14, 15–19, 20–24, 25–29, 30–34, 35–39, 40-44 years), year of arrival (continuous and categorized as 5-year intervals), education level at arrival (0–9 years, 10–12 years, 13+ years, trade certificate/non-university diploma, university degree), marital status at arrival (single, married/common-law, separated/divorced/widowed), official language ability at arrival (English and/or French, neither English or French), refugee status (refugee, non-refugee immigrants [economic and family class immigrants]) and years residing in Ontario (5-year intervals). Residential neighborhood income quintile (1 = lowest to 5 = highest) for the year after arrival was reported in Ontario’s healthcare registry and utilized for analysis. The year after arrival was used to capture residential income quintile for immigrants arriving later in the year.

### Analyses

Population counts and proportions were calculated for year of arrival, age of arrival, education at arrival, income quintile the year after arrival, marital status at arrival, official languages at arrival, refugee status and years residing in Ontario stratified by region of birth.

For all regression analyses unadjusted and adjusted rate ratios (RR and ARR, respectively) were estimated using Poisson regression with the outcome specified as the count of induced abortions and the offset specified as the time in years residing in Ontario since arrival for each female. Crude abortion rates (per 100 PY) and associated 95% CIs were estimated for females from each region and each country if the country population was ≥500 females. Unadjusted and adjusted rate ratios were estimated for region of birth specified as the independent variable and confounders included year of arrival, age at arrival, education at arrival, neighborhood income at arrival and refugee status. Adjusting for year and age at arrival were related to our hypothesis that pre-migration exposures may impact induced abortion after arrival. Specifically, those arriving as adults may be more likely to adhere to the sexual and reproductive health norms related to their country of birth at the time they emigrated. For those arriving younger, sexual and reproductive health norms may be shaped by both the country of origin (through their parents) as well as by the Canadian context.

Persons born in the United States/ Northern & Western Europe/Australia & New Zealand were chosen as the comparator given similar levels of development. Study subjects with missing outcome and covariate data were excluded from regression analyses. We did not adjust for marital status at arrival since this is likely to become misclassified with increasing length of stay, particularly for those arriving younger and single. In secondary analyses, unadjusted and adjusted Poisson regression was conducted for each world region of birth to examine the association between abortion and each of year, age, education, income quintile (at arrival) and refugee status.

Birth order analyses were restricted to mothers who had three or more consecutive singleton births in Ontario between 1993 and 2012. Induced abortion rates (per 100 births) and 95% CIs were estimated for abortions occurring prior to the 1st birth, between the 1st and 2nd births (i.e., prior to the 2nd birth) and between the 2nd and 3rd or higher births (i.e., prior to the third or higher birth) for each region of birth. RRs and 95% CI were also estimated comparing each birth order to that prior to the 1st birth.

## Results

See Table 1a and b for detail on sociodemographic and immigration characteristics by region of birth and supplementary Table S1 for frequencies, proportions and abortion rates (95% CI) by country of birth. In terms of year of arrival, over half of immigrants from the Caribbean arrived between 1991 and 1999 whereas immigrants from South America, West/Middle/East Africa, North Africa/West Asia, East Asia and South Asia mostly arrived in later years. About 30% of immigrants arrived between the ages of 0–19. There was wide variation in the proportion of immigrants who arrived with a post secondary education across regions (between 8 and 50% among those born in the Caribbean and Central Asia, respectively). The region with the highest proportion of refugees was from West/Middle/East Africa (45%), followed by Central America (25%) and North Africa/West Asia (23%). Between 50 and 70% of immigrants from all regions had resided in Ontario for less than 10 years by the end of the follow-up period.

**Table 1:** a: Sociodemographic and immigration characteristics for immigrant females born in the Caribbean, Central America, South America, West/East/Middle Africa, Southern Africa, North Africa/West Asia landing in Ontario (1991–2012) and residing in Ontario for at least one year between 1991 and 2014, [% (n) of column, unless otherwise specified]

	Region of Birth					
	Caribbean	Central America	South America	West/Middle/ East Africa	Southern Africa	North Africa/ West Asia
n females	48,165	16,520	41,197	49,692	3808	66,104
Year of Arrival						
1991–1994	30.2 (14,561)	36.5 (6031)	21.1 (8693)	21.0 (10,439)	25.0 (952)	13.7 (9083)
1995–1999	24.1 (11,602)	16.3 (2691)	19.0 (7831)	19.5 (9669)	26.1 (994)	22.1 (14,625)
2000–2004	18.6 (8954)	16.1 (2659)	23.0 (9483)	23.6 (11,739)	28.3 (1079)	26.8 (17,695)
2005–2009	17.2 (8298)	19.8 (3266)	26.5 (10,934)	23.3 (11,558)	13.6 (517)	24.0 (15,889)
2010–2012	9.9 (4750)	11.3 (1873)	10.3 (4256)	12.7 (6287)	7.0 (266)	13.3 (8812)
Age of Arrival						
0–14	21.4 (10,310)	21.2 (3499)	17.2 (7079)	17.6 (8745)	27.2 (1037)	25.6 (16,940)
15–19	13.8 (6629)	9.9 (1630)	11.0 (4517)	11.9 (5899)	8.2 (312)	10.6 (7010)
20–24	12.2 (5877)	12.7 (2097)	14.0 (5775)	16.6 (8255)	7.4 (283)	13.3 (8765)
25–29	16.1 (7743)	20.0 (3302)	17.9 (7364)	19.9 (9879)	16.2 (618)	16.6 (10,967)
30–34	16.2 (7808)	17.8 (2940)	17.1 (7047)	16.8 (8325)	16.5 (629)	15.0 (9913)
35–39	12.3 (5915)	11.5 (1896)	12.8 (5277)	10.9 (5434)	14.9 (569)	11.4 (7533)
40–44	8.1 (3883)	7.0 (1156)	10.0 (4138)	6.3 (3155)	0.0 (360)	7.5 (4976)
Education at Arrival						
0–9 yrs	34.4 (16,560)	43.4 (7162)	30.6 (12,605)	37.1 (18,450)	32.5 (1236)	42.1 (27,837)
10–12 yrs	31.1 (14,958)	17.0 (2807)	24.8 (10,216)	27.9 (13,887)	18.6 (709)	15.6 (10,337)
13+ yrs	9.9 (4747)	8.9 (1465)	10.1 (4172)	8.2 (4056)	6.7 (256)	6.6 (4392)
trade certificate, non-university diploma	16.8 (8071)	12.4 (2044)	14.3 (5900)	15.0 (7461)	19.8 (755)	8.2 (5416)
bachelors, masters, PhD	7.9 (3829)	18.4 (3042)	20.2 (8304)	11.7 (5838)	22.4 (852)	27.4 (18,122)
Income Quintile (year after arrival)						
1 (lowest)	42.3 (20,369)	39.6 (6534)	38.3 (15,766)	53.0 (26,354)	11.3 (430)	39.8 (26,286)
2	24.1 (11,631)	21.8 (3602)	24.2 (9957)	20.7 (10,270)	13.6 (518)	20.5 (13,557)
3	15.3 (7361)	14.5 (2399)	15.9 (6534)	11.6 (5770)	16.5 (627)	15.9 (10,484)
4	10.2 (4933)	11.3 (1861)	11.4 (4680)	8.2 (4053)	23.8 (906)	12.8 (8449)
5 (highest)	5.5 (2653)	9.7 (1608)	8.3 (3423)	4.3 (2156)	32 (1219)	8.5 (5628)
missing	2.5 (1218)	3.1 (516)	2.0 (837)	2.2 (1089)	0.0 (108)	0.0 (1700)
Marital Status at Arrival						
single	57.2 (27,530)	45.3 (7476)	45.1 (18,598)	51.4 (25,547)	48.6 (1851)	45.5 (30,056)
married, common-law	39.9 (19,241)	51.3 (8471)	52.0 (21,415)	43.6 (21,669)	49.7 (1893)	52.9 (34,972)
separated, divorced, widowed	2.8 (1366)	3.4 (569)	2.9 (1176)	5.0 (2465)	1.7 (64)	1.6 (1065)
missing	0.1 (28)	0.0 (^b^)	0.0 (8)	0.0 (11)	0.0 (0)	0.0 (11)
Official Languages at Arrival						
English and/or French	94.1 (45,338)	65.7 (10,850)	78.7 (32,428)	78.7 (39,116)	97.7 (3719)	62.9 (41,559)
Neither English or French	5.9 (2818)	34.3 (5670)	21.3 (8769)	21.3 (10,575)	2.3 (89)	37.1 (24,544)
missing	0.0 (9)	0.0 (0)	0.0 (0)	0.0 (^b^)	0.0 (0)	0.0 (^b^)
Refugee status						
Refugee	6.5 (3107)	25.2 (4162)	19.8 (8147)	45.4 (22,550)	3.4 (128)	22.9 (15,136)
Non-refugee immigrant ^a^	93.5 (45,058)	74.8 (12,358)	80.2 (33,050)	54.6 (27,142)	96.6 (3680)	77.1 (50,968)
Years residing in Ontario						
0–4 yrs	21.2 (10,194)	22.9 (3778)	25.7 (10,580)	25.6 (12,720)	23.3 (889)	30.6 (20,245)
5–9 yrs	25.7 (12,385)	26.8 (4428)	32.2 (13,267)	29.8 (14,807)	29.8 (1134)	32.4 (21,438)
10–14 yrs	25.7 (12,384)	23.6 (3892)	22.2 (9153)	23.7 (11,778)	28.5 (1084)	22.8 (15,044)
15–19 yrs	20.3 (9754)	17.7 (2931)	14.9 (6152)	15.4 (7636)	14.9 (566)	11.1 (7337)
20+ yrs	7.2 (3448)	9.0 (1491)	5.0 (2045)	5.5 (2751)	3.5 (135)	3.1 (2040)
	Region of Birth					
	Central Asia	East Asia	South Asia	South East Asia & Oceania Islands	Southern & Eastern Europe	U.S. / N&W Europe/ Aus & NZ
n females	3477	151,626	238,697	94,855	105,001	47,551
Year of Arrival						
1991–1994	1.1 (38)	17.3 (26,248)	11.7 (27,978)	24.0 (22,746)	26.3 (27,612)	24.5 (11,658)
1995–1999	19.8 (688)	24.1 (36,586)	21.3 (50,805)	17.1 (16,253)	27.6 (28,993)	23.5 (11,186)
2000–2004	38.1 (1323)	29.4 (44,642)	31.9 (76,238)	18.6 (17,686)	26.3 (27,593)	21.7 (10,342)
2005–2009	30.9 (1073)	20.7 (31,404)	24.1 (57,600)	25.4 (24,124)	14.7 (15,449)	20.9 (9920)
2010–2012	10.2 (355)	8.4 (12,746)	10.9 (26,076)	14.8 (14,046)	5.1 (5354)	9.3 (4445)
Age of Arrival						
0–14	15.9 (554)	14.1 (21,342)	15.9 (38,048)	13.1 (12,451)	19.4 (20,422)	23.5 (11,169)
15–19	9.0 (312)	6.7 (10,106)	9.1 (21,797)	8.4 (7946)	7.8 (8211)	5.9 (2816)
20–24	10.6 (367)	9.9 (15,010)	18.5 (44,138)	11.1 (10,546)	11.6 (12,136)	11.3 (5363)
25–29	16.5 (574)	22.2 (33,607)	21.3 (50,898)	19.2 (18,194)	19.4 (20,403)	20.8 (9896)
30–34	19.0 (661)	20.9 (31,636)	16.2 (38,580)	21.5 (20,421)	18.3 (19,231)	16.6 (7907)
35–39	16.8 (584)	15.7 (23,764)	11.2 (26,790)	16.0 (15,135)	14.2 (14,873)	12.8 (6089)
40–44	12.2 (425)	10.7 (16,161)	7.7 (18,446)	10.7 (10,162)	9.3 (9725)	9.1 (4311)
Education at Arrival						
0–9 yrs	22.0 (764)	26.2 (39,740)	27.3 (65,119)	25.7 (24,420)	28.6 (29,994)	29.1 (13,848)
10–12 yrs	10.4 (363)	16.3 (24,690)	21.5 (51,430)	15.9 (15,043)	16.4 (17,191)	14.0 (6672)
13+ yrs	4.9 (170)	8.3 (12,526)	7.5 (17,813)	7.4 (6994)	7.1 (7504)	9.4 (4478)
trade certificate, non-university diploma	14.0 (486)	18.4 (27,841)	7.0 (16,699)	15.3 (14,542)	16.8 (17,636)	19.5 (9259)
bachelors, masters, PhD	48.7 (1694)	30.9 (46,829)	36.7 (87,636)	35.7 (33,856)	31.1 (32,676)	28.0 (13,294)
Income Quintile (year after arrival)						
1 (lowest)	47.0 (1635)	28.2 (42,777)	42.0 (100,300)	33.5 (31,818)	41.5 (43,535)	16.9 (8038)
2	19.8 (687)	25.9 (39,237)	23.1 (55,212)	21.9 (20,756)	21.8 (22,867)	17.3 (8213)
3	13.3 (461)	18.7 (28,417)	16.6 (39,577)	16.1 (15,227)	13.7 (14,384)	17.5 (8334)
4	9.3 (323)	13.8 (20,903)	10.7 (25,502)	13.0 (12,353)	11.2 (11,770)	19.6 (9325)
5 (highest)	9.0 (314)	11.0 (16,676)	5.5 (13,067)	13.5 (12,833)	9.0 (9451)	26.1 (12,434)
missing	1.6 (57)	2.4 (3616)	2.1 (5039)	2.0 (1868)	2.9 (2994)	2.5 (1207)
Marital Status at Arrival						
single	33.4 (1160)	37.5 (56,822)	35.0 (83,537)	56.3 (53,408)	36.9 (38,747)	43.3 (20,580)
married, common-law	62.5 (2172)	61.1 (92,663)	63.9 (152,462)	41.4 (39,311)	60.1 (63,094)	55.1 (26,198)
separated, divorced, widowed	4.1 (144)	1.4 (2121)	1.1 (2676)	2.2 (2133)	3.0 (3141)	1.6 (772)
missing	0.0 (^b^)	0.0 (20)	0.0 (22)	0.0 (^b^)	0.0 (19)	0.0 (^b^)
Official Languages at Arrival						
English and/or French	57.3 (1991)	45.7 (69,259)	61.7 (147,307)	74.9 (71,086)	52.8 (55,454)	91.1 (43,304)
Neither English or French	42.7 (1486)	54.3 (82,367)	38.3 (91,384)	25.1 (23,767)	47.2 (49,547)	8.9 (4241)
missing	0.0 (0)	0.0 (0)	0.0 (6)	0.0 (^b^)	0.0 (0)	0.0 (6)
Refugee status						
Refugee	14.2 (495)	4.8 (7291)	14.8 (35,338)	2.6 (2489)	16.7 (17,497)	2.5 (1210)
Non-refugee ^a^	85.8 (2982)	95.2 (144,335)	85.2 (203,359)	97.4 (92,366)	83.3 (87,504)	97.5 (46,341)
Years residing in Ontario						
0–4 yrs	29.8 (1037)	23.5 (35,661)	25.2 (60,253)	29.6 (28,113)	18.4 (19,307)	26.8 (12,727)
5–9 yrs	40.1 (1396)	32.7 (49,605)	33.0 (78,853)	30.6 (29,062)	29.8 (31,273)	30.9 (14,703)
10–14 yrs	25.3 (878)	27.9 (42,294)	26.5 (63,184)	21.1 (20,054)	29.7 (31,221)	22.2 (10,533)
15–19 yrs	4.8 (166)	12.7 (19,285)	12.0 (28,607)	14.7 (13,971)	17.0 (17,835)	15.2 (7236)
20+ yrs	0.0 (0)	3.2 (4781)	3.3 (7800)	3.9 (3655)	5.1 (5365)	4.9 (2352)

^a^ includes economic and family class immigrants

^b^ suppressed, count < 5

**Fig. 1:**
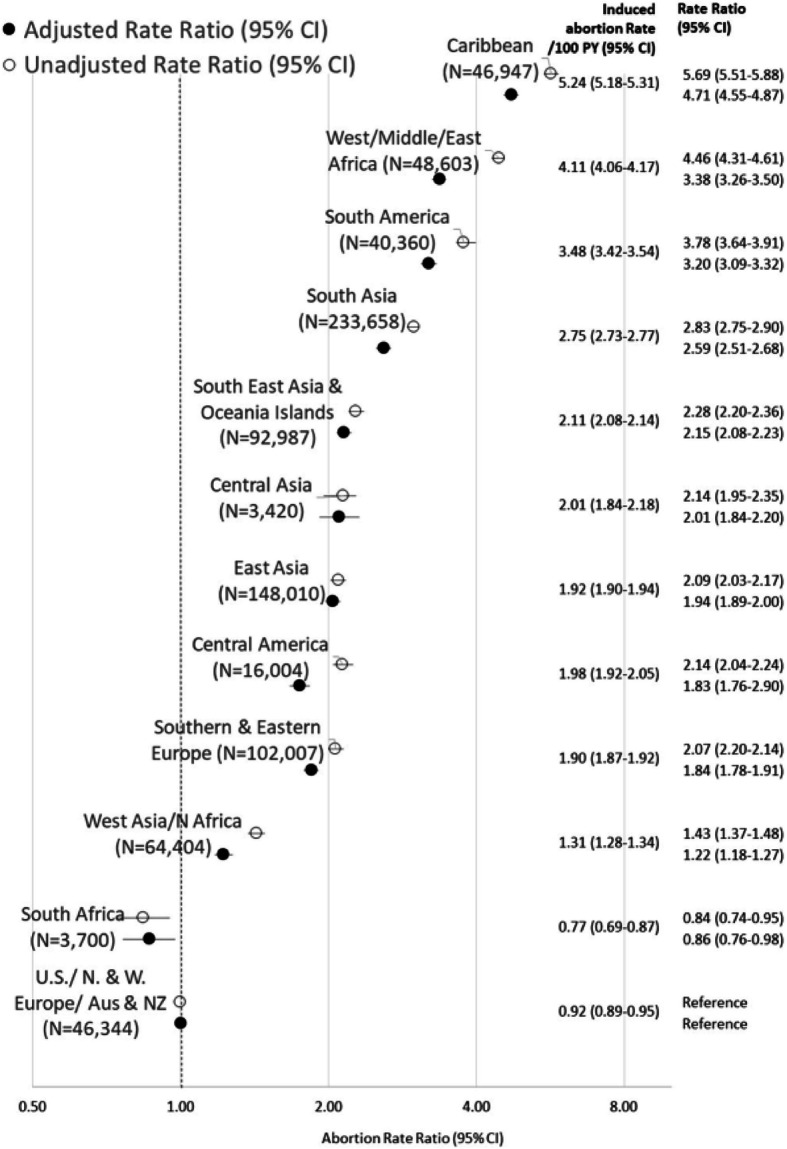
Unadjusted (open circles) and adjusted (black circles) induced abortion rate ratios (RR) and 95% confidence intervals (CI) (1991–2014) by region of birth for immigrant females arriving in Ontario (1991–2012) (*N* = 846,444). Adjusted for year of arrival, age at arrival, education at arrival, neighborhood income quintile at arrival and refugee status

There were 846,444 immigrant females born in the 12 world regions who contributed a total of 7,985,664 person-years (PY) to these analyses (an average of 9.4 years of follow-up per female). The highest abortion rate was among those born in the Caribbean (5.24 per 100 PY, 95% CI 5.51–5.88) and lowest among Southern Africans (0.77, 95% CI 0.69–0.87) (Fig. 1); while the rate among those from the United States/Northern & Western Europe/Australia & New Zealand was 0.92 per 100 PY (95% CI 0.80–0.95) (Ontario rate reported by Statistics Canada in 2005 was 1.19 per 100 women [22]). Those born in the Caribbean were 6 times more likely to have an induced abortion, followed by West/Middle/East Africans and South Americans who were 4 times more likely and South Asians who were 3 times more likely. Those born in South East Asia & Oceania Islands, Central Asia, East Asia, Central America and Southern & Eastern Europe were twice as likely to have an induced abortion, while those from North Africa/West Asia were 31% more likely to have an induced abortion. In secondary analyses (supplementary Fig. S2a-c including a short interpretation) the association between abortion rates and characteristics at arrival stratified by region of birth were also examined.

**Fig. 2:**
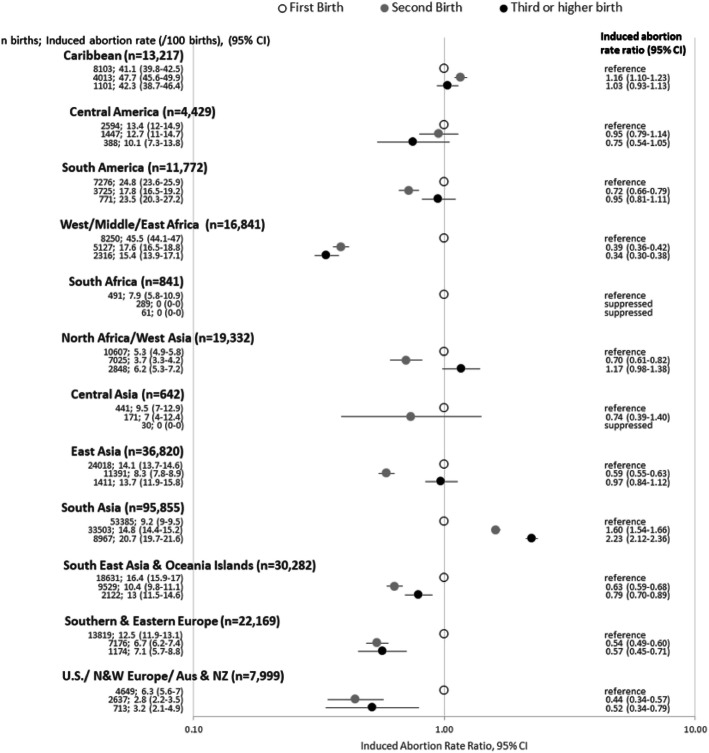
Prior induced abortion rate ratios (95% CI) by birth order and region of birth among immigrant females who had up to three consecutive births in Ontario (1993–2014) (*N* = 152,264)

Of the immigrants included in the above described cohort, 152,264 had three or more consecutive births in Ontario (Fig. 2). Those born in West/Middle/East Africa, South East Asia & Oceania Islands, Southern & Eastern Europe and United States/Northern & Western Europe/Australia & NZ were all less likely to have an induced abortion prior to the 1st birth vs. after the 1st birth, while only South Asians were more likely to have an abortion after the 1st birth (prior to both 2nd and 3rd births). Abortion rates did not differ significantly by birth order among Central Americans; while among those from the Caribbean abortion rates remain high and did not vary as strongly by birth order compared to other regions. South Americans, North Africans/West Asians and East Asians were less likely to have an abortion prior to their 2nd birth but more likely or as likely to have an abortion prior to their 3rd birth. Abortion counts for Southern Africans and Central Asians were too small to be reported and abortion rates were not estimated. The pattern was mixed for those from the remaining regions, with some estimates indicating no difference in abortion rates by birth order.

## Discussion

In this large population-based study in Ontario, we found that immigrant females born in all world regions, except Southern Africa, were 2–5 times more likely to have an induced abortion compared to females born in the United States/Northern & Western Europe/Australia & New Zealand after adjustment for sociodemographic and immigration characteristics. Comparing to other published data [22] immigrants from most regions in our study had higher abortions rates than the general Ontario population (1.19 per 100). Ours is one of a handful of studies examining abortion among immigrants residing in high-income countries [23–29]. Smaller studies with less diverse populations of immigrants from Finland [23] and Norway [29] also found higher rates of induced abortion among immigrants compared to non-immigrants. In our study, immigrants from most world-regions were less likely to have an abortion after the 1st birth compared to prior to the 1st birth, with more variability prior to the 3rd birth. A Norwegian study found the opposite, with higher abortion rates after the 1st child compared to before the 1st child (not stratified by region of birth) [29].

Given that the majority of abortions result from unintended pregnancies [1], our findings suggest unmet need for contraception varies considerably by region of birth among immigrants in Ontario. Multi-country and Canadian studies describing contraceptive use potentially provide insight into reasons for this unmet need. According to a Demographic and Health Survey (DHS) [30] married women wanting to avoid pregnancy did not use contraception because: i) of concerns about the health risks of modern contraceptives, ii) someone close to them opposed contraception use and iii) of the misperception that infrequent sex, breastfeeding or not resuming menstruation after birth, protects against pregnancy. Among never-married women wanting to avoid pregnancy, infrequent sex, concerns about contraceptive side-effects and not being married were cited for not using contraception. As Mengesha et al. [13] suggests, such pre-migration knowledge and experiences related to sexual and reproductive health may persist post-migration. In fact, a Canadian abortion clinic study [31] is consistent with the DHS study in that more frequent use of less effective contraceptives among immigrant (*n* = 533) compared to non-immigrant (*n* = 466) women was linked to health concerns of hormonal contraceptives.

There are two examples that may help to further elucidate the connection between pre- and post-migration sexual and reproductive health contexts. A review of the factors influencing contraceptive use in Sub-Saharan Africa [32] (SSA) indicate women experience immense societal pressure to bear children and have concerns that modern contraception harms fertility. However once fertility is proven by bearing children, a woman can “maintain her husband’s respect and stabilize their relationship” and adopt a contraceptive method going forward, if the husband approves. These societal expectations may help explain why females from West/Middle/East Africa in our study had the 2nd highest overall abortion rate among all regions and more specifically why the abortion rate was highest prior to the 1st birth and dropped significantly for subsequent births. In South Asia early marriage, lower female autonomy and limited participation in personal health care decisions have been described [33] which limits access to effective family planning and contraceptive counseling. These circumstances carrying over to the post-migration context may help explain why South Asians had the 4th highest abortion rate of all regions and were the only group to be more likely to have an abortion *after* both their 2nd and 3rd births. A related phenomenon, is that of sex-selective abortion, an extreme manifestation of son preference stemming from specific forms of patriarchy (i.e., patrilocality and patrilineality) [34] which may help explain higher abortion rates after the 1st birth in our study. A study led by co-author MLU demonstrated that son-biased sex ratios, particularly after two previous daughters, was associated with abortion among immigrants from India [18] (42% of South Asian immigrant females in Ontario – see supplementary Table S1).

The post-migration health care context is also very likely to impact induced abortion and unmet needs for contraception among immigrant women. A systematic review from Australia identified numerous barriers and facilitators to sexual and reproductive health care affecting immigrant women that are likely relevant to Canada given similarities in terms of a high proportion of immigrants and publicly funded health care. Barriers included a healthcare system and model of care which were not culturally responsive, the sensitive and culturally bound nature of sexual and reproductive health limiting some women’s ability to freely discuss and accept medical advice; language and communication; and difficulty navigating the health care system. Facilitators included being provided with a female health professional, having health professionals that listened to concerns, answered questions and explained options [13]. A known barrier in the Canadian context, is that immigrants residing in Ontario for as long as 10+ years have lower access to enhanced primary care models, particularly those which are patient-centered, comprehensive, coordinated and continuous [12, 35] which make effective contraception counseling and ongoing family planning less accessible. Finally, due to regulatory barriers, access to long-acting reversible contraceptives (LARCs) specifically all types of subdermal implants and many types of intrauterine devices (IUDs) are not available in Canada [36, 37]. IUDs that are available have large upfront costs and require and appointment with a doctor trained in insertion as well as time off work for the procedure and to rest. In addition, non-hormonal IUDs may not be covered by drug plans [38].

### Potential strategies for reducing inequity

Our findings provide information to further target recommended Canadian immigrant clinical care guidelines for screening for unmet need and contraceptive counseling [39]. To improve access, it also may be necessary to increase the scope of practitioners providing counseling and contraception (e.g., nurses with a directive). Training in the provision of culturally humble [40] care is likely critical, given the importance of socio-cultural context for the sexual and reproductive health of immigrants [11]. Cultural humility moves beyond cultural competency, criticized for reinforcing harmful cultural and racial stereotypes, to encourage clinicians to engage in a process of self-reflection aiming to build honest and trusting relationships with patients. For Caribbean and West/Middle/East African populations who commonly experience anti-Black racism in the Canadian health care system [41, 42] which may prevent or delay seeking sexual and reproductive health care, a culturally humble approach may be particularly important. Improving availability and access to high-quality, professional interpretation [43, 44] is also important. Overcoming regulatory barriers to LARCs [38] and providing a universal contraception subsidy [36, 37], given the high costs of contraception and the high proportion of immigrants with low incomes, may increase opportunities for immigrants to access and utilize contraception that best suits a variety of needs and circumstances. Finally, improving immigrant’s access to high quality primary care [12] is critical to establish and enable ongoing contraceptive counseling and family planning.

### Limitations and strengths

To our knowledge, this study includes the largest and most diverse population of immigrants residing in a high-income country eligible for publicly funded health care. We used population-based health care databases to identify females who underwent induced abortions and therefore did not rely on self-reported surveys which suffer from underreporting. We were unable to examine types of contraception use among immigrants which could provide insight into associations with abortion. We could not capture a small number of abortions provided in private clinics since they are not subject to the same reporting requirements. We were also unable to exclude a small proportion of abortions which were health related or medically indicated (< 4%) [20] since there is no requirement to provide a reason for abortion. We recognize that there is considerable heterogeneity within regions exemplified by country-level abortion rates within regions (see Table S1) resulting from differing social, cultural, religious and systemic determinants of abortion which we were unable to disentangle with the data we had. The advantage of examining abortion rates by these regions is that it facilitated direct comparison to global health studies of abortion, contraceptive prevalence and unmet need [10, 30] which allowed us to shed light on the pre-migration sexual and reproductive health care context. Given that we used administrative data, we were unable to provide a direct explanation of why induced abortion rates vary by region of birth and birth order. However, we looked to relevant literature from other high-income countries, Canada and the global health context to provide some insight. We could not identify migrants with temporary work permits (typically lasting 1 year) in our databases even though most are eligible for provincial health care. We also could not examine induced abortion among a small number of undocumented migrants (estimated at ~ 1/2 million in all of Canada [16]), given that these individuals are not eligible for publicly funded health care.

## Conclusion

Our study finds that there were high rates of induced abortion among immigrants now residing in Ontario particularly for those from the Caribbean, West/Middle/East Africa and South America regardless of birth order; and from South Asia after the 1st birth and from most other regions prior to the 1st birth. Suggested strategies to reduce these inequities and unintended pregnancies will likely benefit immigrant and Canadian-born persons alike.

## Supplementary information

**Additional file 1: Table S1.** Frequency, proportion and abortion rates (/100 PY) (95% CI) (1991–2014) by immigrant female’s countries of birth (1991–2012) (where country counts ≥500 only) and legal status of abortion in birthplace. **Figure S2a.** Unadjusted (open circles) and adjusted (closed circles) induced abortion rate ratios (RR) and 95% confidence intervals (CI) (1991–2014) for sociodemographic factors among immigrant females born in the Caribbean, Central America, South America and West/East/Middle Africa arriving in Ontario (1991–2012). Models adjusted for year, age, education, neighborhood income quintile and refugee status (adjusted for variable when not examined as the main sociodemographic exposure of interest). **b**: Unadjusted (open circles) and adjusted (black circles) induced abortion rate ratios (RR) and 95% confidence intervals (CI) (1991–2014) for sociodemographic factors among immigrant females born in South Africa, North Africa/West Asia, Central Asia and East Asia arriving in Ontario (1991–2012) and residing in Ontario for at least one year. Models adjusted for year, age, education, neighborhood income quintile and refugee status (adjusted for variable when not examined as the main sociodemographic exposure of interest). **c**: Unadjusted (open circles) and adjusted (black circles) induced abortion rate ratios (RR) and 95% confidence intervals (CI) (1991–2014) for sociodemographic factors among immigrant females born in South Asia, South-East Asia & Oceania Islands, Southern & Eastern Europe and United States/Northern & Western Europe/Australia & New Zealand arriving in Ontario (1991–2012) and residing in Ontario for at least one year. Models adjusted for year, age, education, neighborhood income quintile and refugee status (adjusted for variable when not examined as the main sociodemographic exposure of interest).

## Data Availability

The dataset from this study is held securely in coded form at ICES. While data sharing agreements prohibit ICES from making the dataset publicly available, access may be granted to those who meet pre-specified criteria for confidential access, available at www.ices.on.ca/DAS. The full dataset creation plan and underlying analytic code are available from the authors upon request, understanding that the computer programs may rely upon coding templates or macros that are unique to ICES and are therefore either inaccessible or may require modification.

## Abbreviations

ARR: Adjusted rate ratio
CIHI: Canadian Institute for Health Information
DAD: Discharge Abstract Database
IRCC-PRD: Immigration and Refugee Citizenship Canada Permanent Resident Database
NACRS: National Ambulatory Care Reporting System
OHIP: Ontario Health Insurance Plan
ORGD: Office of the Registrar General’s Vital Statistics Database
PY: Person-years
RR: Rate ratio
