# Supplementary material for: Induced abortion according to immigrants’ birthplace: a population-based cohort study
Source: Reprod Health. 2020 Sep 14;17:143. doi: 10.1186/s12978-020-00982-z (PMC7488678; doi:10.1186/s12978-020-00982-z)
Supplement: Supplementary file 1 — Additional file 1: Table S1. Frequency, proportion and abortion rates (/100 PY) (95% CI) (1991–2014) by immigrant female’s countries of birth (1991–2012) (where country counts ≥500 only) and legal status of abortion in birthplace. Figure S2a. Unadjusted (open circles) and adjusted (closed circles) induced abortion rate ratios (RR) and 95% confidence intervals (CI) (1991–2014) for sociodemographic factors among immigrant females born in the Caribbean, Central America, South America and West/East/Middle Africa arriving in Ontario (1991–2012). Models adjusted for year, age, education, neighborhood income quintile and refugee status (adjusted for variable when not examined as the main sociodemographic exposure of interest). b: Unadjusted (open circles) and adjusted (black circles) induced abortion rate ratios (RR) and 95% confidence intervals (CI) (1991–2014) for sociodemographic factors among immigrant females born in South Africa, North Africa/West Asia, Central Asia and East Asia arriving in Ontario (1991–2012) and residing in Ontario for at least one year. Models adjusted for year, age, education, neighborhood income quintile and refugee status (adjusted for variable when not examined as the main sociodemographic exposure of interest). c: Unadjusted (open circles) and adjusted (black circles) induced abortion rate ratios (RR) and 95% confidence intervals (CI) (1991–2014) for sociodemographic factors among immigrant females born in South Asia, South-East Asia & Oceania Islands, Southern & Eastern Europe and United States/Northern & Western Europe/Australia & New Zealand arriving in Ontario (1991–2012) and residing in Ontario for at least one year. Models adjusted for year, age, education, neighborhood income quintile and refugee status (adjusted for variable when not examined as the main sociodemographic exposure of interest). [file 12978_2020_982_MOESM1_ESM.docx]

Supplementary Tables & Figures

Table S1: Frequency, proportion and abortion rates (/100 PY) (95% CI) (1991-2014) by immigrant female’s countries of birth (1991-2012) (where country counts ≥ 500 only) and legal status of abortion in birthplace

| **Region of birth**  Country of birth | N females | % of females within region | Abortion rate (/100 PY), 95% CI | Legal status of abortion in country of birth^a^ |
| --- | --- | --- | --- | --- |
| **Caribbean** | **48,165** | **100.0%** | **5.69 (5.51-5.88)** |  |
| Jamaica | 21,875 | 45.4% | 6.01 (5.91-6.10) | 4 |
| Trinidad & Tobago, Republic of | 10,961 | 22.8% | 4.03 (3.92-4.13) | 4 |
| Cuba | 3,546 | 7.4% | 3.72 (3.51-3.94) | 6 |
| Grenada | 2,544 | 5.3% | 6.84 (6.53-7.16) | 3 |
| Haiti | 2,483 | 5.2% | 4.42 (4.10-4.76) | 1 |
| St. Vincent and the Grenadines | 2,318 | 4.8% | 6.55 (6.20-6.90) | 5 |
| Dominican Republic | 1,410 | 2.9% | 3.55 (3.21-3.92) | 1 |
| St. Lucia | 1,388 | 2.9% | 5.45 (5.04-5.90) | 4 |
| Barbados | 940 | 2.0% | 4.05 (3.69-4.45) | 5 |
| Dominica | 532 | 1.1% | 5.42 (4.87-6.04) | 2 |
| **Central America** | **16,520** | **100.0%** | **2.14 (2.04-2.24)** |  |
| Mexico | 6,707 | 40.6% | 1.11 (1.02-1.19) | 2 |
| El Salvador | 4,488 | 27.2% | 2.26 (2.14-2.38) | 1 |
| Guatemala | 1,838 | 11.1% | 2.35 (2.15-2.56) | 2 |
| Nicaragua | 1,575 | 9.5% | 2.47 (2.26-2.68) | 1 |
| Costa Rica | 754 | 4.6% | 2.53 (2.17-2.94) | 3 |
| Honduras | 741 | 4.5% | 3.07 (2.71-3.49) | 1 |
| Guyana | 13,774 | 83.4% | 5.64 (5.53-5.76) | 6 |
| **South America** | **47,197** | **100.0%** | **3.78 (3.64-3.91)** |  |
| Colombia | 9,956 | 21.1% | 1.41 (1.32-1.49) | 4 |
| Brazil | 4,692 | 9.9% | 1.15 (1.05-1.26) | 2 |
| Peru | 2,825 | 6.0% | 3.16 (2.95-3.38) | 3 |
| Ecuador | 2,588 | 5.5% | 3.03 (2.83-3.24) | 3 |
| Venezuela | 2,365 | 5.0% | 1.72 (1.55-1.91) | 2 |
| Argentina | 2,354 | 5.0% | 1.96 (1.79-2.15) | 3 |
| Chile | 1,272 | 2.7% | 2.31 (2.07-2.58) | 2 |
| Uruguay | 661 | 1.4% | 2.48 (2.14-2.87) | 6 |
| **West/Middle/East Africa** | **49,692** | **100.0%** | **4.46 (4.31-4.61)** |  |
| Somalia, Democratic Republic of | 11,016 | 22.2% | 1.86 (1.79-1.93) | 2 |
| Ethiopia | 6,963 | 14.0% | 5.77 (5.61-5.94) | 3 |
| Nigeria | 6,833 | 13.8% | 4.29 (4.11-4.47) | 2 |
| Ghana | 6,313 | 12.7% | 7.07 (6.88-7.27) | 4 |
| Kenya | 3,169 | 6.4% | 2.33 (2.15-2.51) | 3 |
| Congo, Democratic Republic of | 2,438 | 4.9% | 5.32 (4.99-5.66) | 1 |
| Zimbabwe | 1,896 | 3.8% | 4.63 (4.27-5.00) | 3 |
| Tanzania, United Republic of | 1,396 | 2.8% | 2.24 (1.99-2.51) | 2 |
| Mauritius | 1,286 | 2.6% | 1.47 (1.26-1.72) | 4 |
| Eritrea | 1,183 | 2.4% | 4.92 (4.47-5.41) | 4 |
| Uganda | 1,002 | 2.0% | 3.87 (3.47-4.31) | 2 |
| Burundi | 948 | 1.9% | 5.59 (5.06-6.16) | 3 |
| Rwanda | 712 | 1.4% | 4.80 (4.24-5.42) | 3 |
| Cameroon, Federal Republic | 679 | 1.4% | 4.52 (3.92-5.21) | 3 |
| Angola | 544 | 1.1% | 4.93 (4.32-5.61) | 1 |
| Sierra Leone | 525 | 1.1% | 7.09 (6.37-7.88) | 4 |
| **Southern Africa** | **3,808** | **100.0%** | **0.84 (0.74-0.95)** |  |
| South Africa, Republic of | 3,635 | 95.5% | 0.71 (0.63-0.80) | 6 |
| **North Africa/West Asia** | **66,104** | **100.0%** | **1.43 (1.37-1.48)** |  |
| Iraq | 13,234 | 20.0% | 1.40 (1.33-1.47) | 1 |
| Egypt | 7,895 | 11.9% | 0.61 (0.56-0.67) | 1 |
| Lebanon | 7,375 | 11.2% | 0.91 (0.85-0.98) | 2 |
| Turkey | 4,745 | 7.2% | 1.97 (1.84-2.10) | 6 |
| Saudi Arabia | 4,449 | 6.7% | 1.18 (1.07-1.29) | 3 |
| United Arab Emirates | 3,800 | 5.7% | 1.06 (0.95-1.10) | 2 |
| Kuwait | 3,688 | 5.6% | 0.90 (0.80-1.00) | 3 |
| Sudan, Democratic Republic of | 3,372 | 5.1% | 2.86 (2.68-3.05) | 2 |
| Syria | 3,233 | 4.9% | 1.46 (1.33-1.61) | 2 |
| Israel | 2,954 | 4.5% | 1.11 (0.98-1.20) | 4 |
| Jordan | 2,834 | 4.3% | 0.75 (0.65-0.87) | 3 |
| Morocco | 1,278 | 1.9% | 2.74 (2.44-3.06) | 3 |
| Libya | 1,037 | 1.6% | 0.67 (0.52-0.87) | 2 |
| Palestinian Authority | 887 | 1.3% | 0.85 (0.65-1.10) | 2 |
| Algeria | 863 | 1.3% | 1.11 (0.88-1.30) | 4 |
| Azerbijan | 828 | 1.3% | 3.32 (2.89-3.81) | 6 |
| Armenia | 702 | 1.1% | 2.03 (1.68-2.46) | 6 |
| Yemen | 578 | 0.9% | 1.31 (1.02-1.67) | 2 |
| Georgia | 540 | 0.8% | 2.60 (2.13-3.17) | 6 |
| Qatar | 524 | 0.8% | 0.80 (0.57-1.10) | 3 |
| Bahrain | 517 | 0.8% | 1.74 (1.41-2.16) | 6 |
| **Central Asia** | **3,477** | **100.0%** | **2.14 (1.95-2.35)** |  |
| Kazakhstan | 1,735 | 49.9% | 1.98 (1.76-2.22) | 6 |
| Uzbekistan | 1,022 | 29.4% | 2.14 (1.83-2.50) | 6 |
| **East Asia** | **151,262** | **100.0%** | **2.09 (2.03-2.17)** |  |
| China, People's Republic of | 98,261 | 65.0% | 2.46 (2.43-2.49) | 6 |
| Hong Kong | 26,996 | 17.8% | 1.00 (0.97-1.00) | 5 |
| Korea, Republic | 16,246 | 10.7% | 1.35 (1.29-1.41) | 3 |
| Taiwan | 5,299 | 3.5% | 1.30 (1.21-1.40) | 5 |
| Japan | 3,944 | 2.6% | 0.61 (0.53-0.69) | 5 |
| Macao | 543 | 0.4% | 1.40 (1.13-1.74) | unknown |
| **South Asia** | **238,697** | **100.0%** | **2.83 (2.75-2.90)** |  |
| India | 100,990 | 42.3% | 3.27 (3.23-3.30) | 5 |
| Pakistan | 51,022 | 21.4% | 1.45 (1.41-1.48) | 3 |
| Sri Lanka | 34,607 | 14.5% | 3.73 (3.66-3.78) | 2 |
| Iran | 25,581 | 10.7% | 2.15 (2.09-2.21) | 2 |
| Bangladesh | 13,149 | 5.5% | 2.18 (2.09-2.26) | 2 |
| Afghanistan | 10,843 | 4.5% | 2.14 (2.02-2.19) | 2 |
| Nepal | 2,305 | 1.0% | 3.03 (2.74-3.34) | 6 |
| **South-East Asia & Oceania Islands** | **94,855** | **100.0%** | **2.28 (2.75-2.90)** |  |
| Philippines | 71,375 | 75.2% | 1.86 (1.83-1.89) | 1 |
| Vietnam, Socialist Republic of | 13,829 | 14.6% | 3.12 (3.03-3.20) | 6 |
| Malaysia | 1,979 | 2.1% | 1.55 (1.39-1.73) | 4 |
| Indonesia | 1,741 | 1.8% | 0.82 (0.68-0.97) | 2 |
| Thailand | 1,548 | 1.6% | 1.31 (1.12-1.52) | 4 |
| Cambodia | 1,399 | 1.5% | 2.72 (2.47-3.00) | 6 |
| Myanmar | 1,010 | 1.1% | 2.06 (1.76-2.42) | 2 |
| Singapore | 905 | 1.0% | 1.01 (0.82-1.20) | 6 |
| Fiji | 575 | 0.6% | 5.16 (4.67-5.69) | 5 |
| **Southern & Eastern Europe** | **105,001** | **100.0%** | **2.07 (2.20-2.14)** |  |
| Poland | 17,500 | 16.7% | 1.38 (1.33-1.42) | 3 |
| Romania | 14,402 | 13.7% | 2.25 (2.18-2.33) | 6 |
| Russia | 13,804 | 13.1% | 1.95 (1.88-2.03) | 6 |
| Ukraine | 11,632 | 11.1% | 2.13 (2.04-2.22) | 6 |
| Yugoslavia | 11,498 | 11.0% | 2.06 (1.99-2.13) | unknown |
| Union of Soviet Socialist Republics | 4,559 | 4.3% | 2.52 (2.38-2.65) | unknown |
| Albania | 4,379 | 4.2% | 2.36 (2.21-2.53) | 6 |
| Bosnia-Hercegovina | 4,359 | 4.2% | 2.00 (1.88-2.13) | 6 |
| Portugal | 4,048 | 3.9% | 1.58 (1.48-1.69) | 6 |
| Bulgaria | 3,702 | 3.5% | 1.66 (1.53-1.79) | 6 |
| Hungary | 2,579 | 2.5% | 2.26 (2.08-2.44) | 6 |
| Belarus | 2,010 | 1.9% | 1.94 (1.74-2.16) | 6 |
| Moldova | 1,654 | 1.6% | 2.13 (1.90-2.39) | 6 |
| Croatia | 1,624 | 1.5% | 1.55 (1.38-1.73) | 6 |
| Macedonia | 1,188 | 1.1% | 1.88 (1.64-2.16) | 6 |
| Slovak Republic | 1,186 | 1.1% | 0.98 (0.81-1.10) | 6 |
| Italy | 1,120 | 1.1% | 0.95 (0.78-1.10) | 6 |
| Czech Republic | 1,045 | 1.0% | 2.72 (2.43-3.05) | 6 |
| Greece | 583 | 0.6% | 1.36 (1.09-1.68) | 6 |
| Czechoslovakia | 544 | 0.5% | 1.57 (1.32-1.87) | 6 |
| Serbia and Montenegro | 535 | 0.5% | 1.52 (1.14-2.02) | 6 |
| **US, N. & W. Europe, Australia & NZ** | **47,551** | **100.0%** | **0.92 (0.89-0.95)** |  |
| United States of America | 18,223 | 38.3% | 0.71 (0.67-0.75) | 6 |
| United Kingdom and Colonies | 14,530 | 30.6% | 1.12 (1.07-1.17) | 5 |
| Germany, Federal Republic of | 3,875 | 8.1% | 1.06 (0.96-1.10) | 6 |
| France | 1,813 | 3.8% | 0.80 (0.67-0.94) | 6 |
| Netherlands | 1,684 | 3.5% | 0.55 (0.45-0.68) | 6 |
| Australia | 1,458 | 3.1% | 0.46 (0.36-0.58) | 6 |
| Ireland | 1,044 | 2.2% | 0.86 (0.71-1.00) | 2 |
| Switzerland | 804 | 1.7% | 0.80 (0.63-1.00) | 6 |
| Latvia | 674 | 1.4% | 1.84 (1.53-2.20) | 6 |
| Lithuania | 674 | 1.4% | 1.62 (1.34-1.97) | 6 |

^a^ Legal status of abortion in country of origin as reported in Abortion Worldwide, 2017 (Guttmacher Institute, 2018);

1 – prohibited altogether (no explicit level exception)

2 – to save life of woman

3 – to save life of woman/preserve physical health

4 - to save life of woman/preserve physical health/mental health

5 - to save life of woman/preserve physical health/mental health/on socioeconomic grounds

6 – no restriction as to reason (with gestational and other requirements)

Figure S2a: Unadjusted (open circles) and adjusted (closed circles) induced abortion rate ratios (RR) and 95% confidence intervals (CI) (1991-2014) for sociodemographic factors among immigrant females born in the Caribbean, Central America, South America and West/East/Middle Africa arriving in Ontario (1991-2012). Models adjusted for year, age, education, neighborhood income quintile and refugee status (adjusted for variable when not examined as the main sociodemographic exposure of interest).


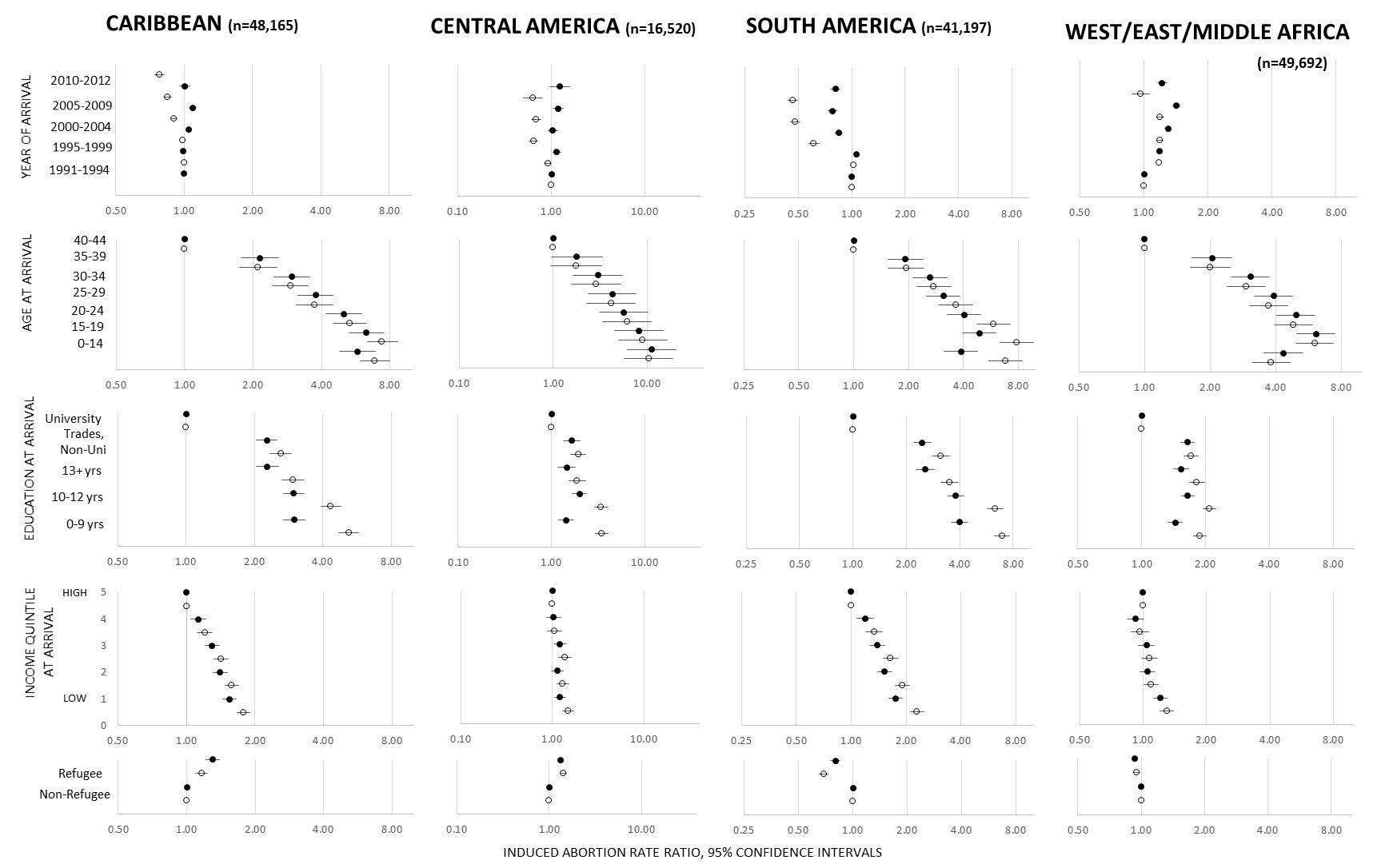


Figure S2b: Unadjusted (open circles) and adjusted (black circles) induced abortion rate ratios (RR) and 95% confidence intervals (CI) (1991-2014) for sociodemographic factors among immigrant females born in South Africa, North Africa/West Asia, Central Asia and East Asia arriving in Ontario (1991-2012) and residing in Ontario for at least one year. Models adjusted for year, age, education, neighborhood income quintile and refugee status (adjusted for variable when not examined as the main sociodemographic exposure of interest).


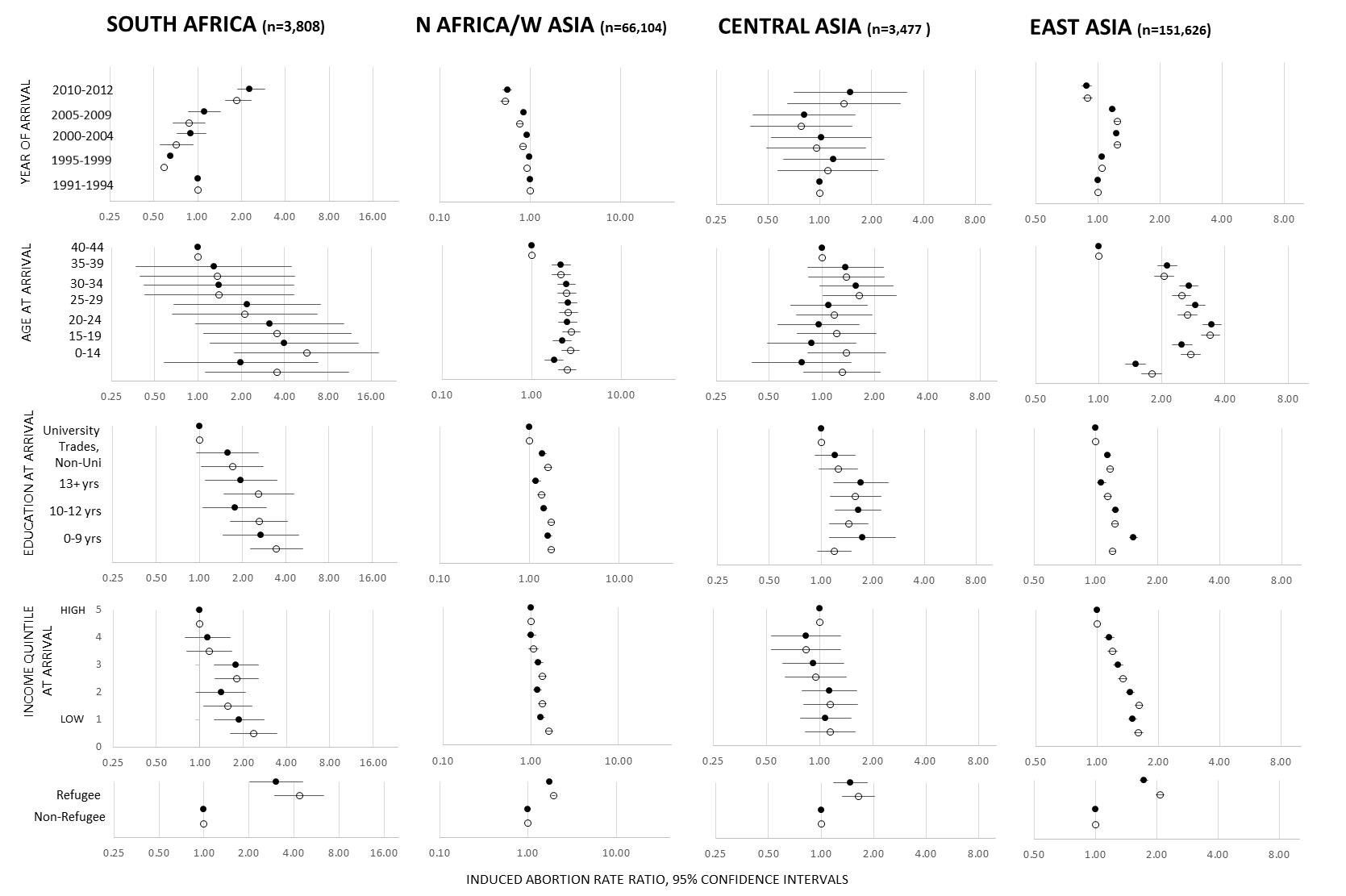


Figure S2c: Unadjusted (open circles) and adjusted (black circles) induced abortion rate ratios (RR) and 95% confidence intervals (CI) (1991-2014) for sociodemographic factors among immigrant females born in South Asia, South-East Asia & Oceania Islands, Southern & Eastern Europe and United States/Northern & Western Europe/Australia & New Zealand arriving in Ontario (1991-2012) and residing in Ontario for at least one year. Models adjusted for year, age, education, neighborhood income quintile and refugee status (adjusted for variable when not examined as the main sociodemographic exposure of interest).


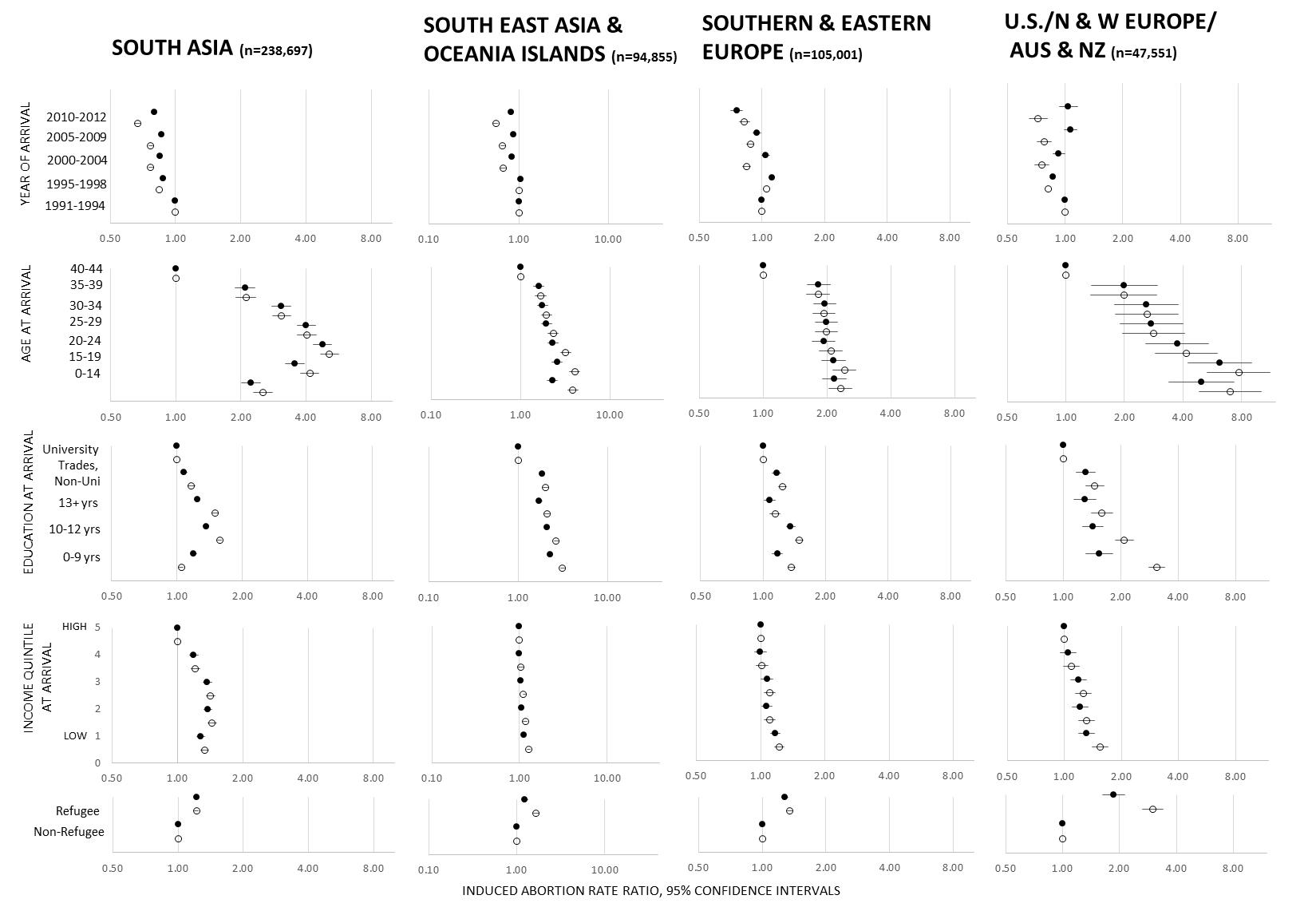


Interpretation of Figures S2a-S2c

For most regions, after adjustment, recently arrived cohorts experienced either similar or increased rates of abortion compared to those who arrived between 1991-1994. Those born in almost all regions exhibited the highest abortion rates at young ages at arrival (<19), with the exception of those born in East Asia and South Asia who experienced the highest rates when arriving between the ages of 20-24. For all regional groups, those arriving between the ages of 0-14 either had lower (West/East/Middle Africa, East Asia, South Asia) or similar abortion rates compared to those arriving between 15-19 years of age. Generally, those with lower education at arrival (0-12 years) had higher abortion rates compared to those with a University education; with the notable exception of those born in South Asia arriving with 0-9 years of education who had a significantly lower abortion rate compared to those with a University education. A decreasing trend in abortion rates with education was most evident for those born in Caribbean, South America and U.S./N & W Europe/Aus & NZ. Those residing in the lowest income neighborhood had higher abortion rates compared to those residing in the highest income neighborhoods. Year, age, education and income quintile were not as strongly associated with abortion rates for those born in North Africa /West Asia as compared to other regions. Refugees born in all regions, except South America and West/Middle/East Africa, had higher abortion rates than their non-refugee immigrant counterparts.
